# Coping efforts made: Psychological burden of people living with tuberculosis due to social stigma in society. A qualitative phenomenology study

**DOI:** 10.1371/journal.pone.0303331

**Published:** 2024-07-30

**Authors:** Abd Nasir, Intan Idiana Hassan, Anwar Ma’ruf, Novianto Edi Suharno, Sianiwati Goenharto, Cucuk Rahmadi Purwanto, Anestasia Pangestu Mei Tyas

**Affiliations:** 1 Faculty of Vocational Studies, Universitas Airlangga, Surabaya, Indonesia; 2 School of Health Sciences, Universiti Sains Malaysia, Malaysia; 3 Department of Basic Veterinary Science, Faculty of Veterinary Medicine, Universitas Airlangga Surabaya, Surabaya, Indonesia; 4 Doctoral Candidate of Faculty of Nursing, Universitas Airlangga, Surabaya, Indonesia; 5 Faculty of Vocational Studies, Airlangga University, Surabaya, Indonesia; Kilimanjaro Clinical Research Institute, UNITED REPUBLIC OF TANZANIA

## Abstract

The psychological burden is greatly felt by people living with tuberculosis because the characteristics of the disease are very visible and very contagious, and the obligation to take the right dose of medication with long treatment. This is what makes tuberculosis a very stigmatic disease. The aim of this research is to explore the psychological burden felt by people living with tuberculosis due to social stigma by society and how coping efforts are made. This research uses a qualitative phenomenological design through in-depth face-to-face interviews which take place in a semi-structured manner with the hope of obtaining complete data. The purposive sampling method was used in this research with Participatory Interpretative Phenomenology analysis involving 25 participants consisting of 16 men and 9 women. This research produced several themes, including 1) "The Perception of stigma limiting space and time", 2) "The Opportunities for interpersonal interaction become narrow", 3) "The mental stress as a challenging emotion", and 4) " Expanding coping efforts”. The psychological burden is felt by people living with tuberculosis because society’s treatment is felt to be very discriminatory due to the social stigma that has developed in society so they lose the opportunity to interact with society. For that reason, they tried to explore some of the personal and environmental resources used to modify adaptive coping in resolving perceived psychological burdens. Given the possibility of ongoing stigma and discrimination during tuberculosis treatment programs, it is important to consider the psychological burden in this context, both on the general population and on groups affected by stigma.

## Introduction

Tuberculosis is characterized as a disease with long treatment and requires precise dosage to be adhered to, thus placing pharmacological management as the main target [1]. Meanwhile, a prolonged cough with typical sputum production [2, 3], and poor nutritional status [4–6], really characterizes their existence. That is why, tuberculosis is treated very differently by society [7], so that psychological burdens are part of their lives [8, 9], and depression is increasingly worsening the condition [10], thus playing an active role in increasing tuberculosis cases [11]. The global tuberculosis report estimates that 9.9 million people are infected with TB globally, and 1,514,000 people died from TB in 2020 and places Indonesia in 4th position in the world after Brazil, China, and India with the discovery of 40,000 patients [12].

Psychological burden is a tiring emotional experience felt by people living with tuberculosis [13]. The stigma of tuberculosis as a highly contagious infectious disease [14, 15], prevents them from actively engaging in social contact, especially if there is a desire to control the situation, both what has happened and what will happen. This is because society treats them indiscriminately [16]. Several studies have raised concerns about the impact of tuberculosis stigma on mental health, and psychological issues are an important theme in these discussions [17]. The stigma and discrimination that has developed in society towards people with tuberculosis has also been proven to contribute to an increase in drug withdrawal rates [18], a decrease in the number of new cases discovered [19], and a decrease in motivation to seek treatment definitive [20]. This is because they are significantly disrupted by normal patterns of social interaction, which has the potential to place enormous stress on their mental health [21]. Therefore, increasing our understanding of the psychosocial impact of stigma on people living with tuberculosis can provide input for the development of strategies to reduce the potential negative impact in responding to the stigma that has become part of their lives, now and in the future.

Stigma in social interaction describes an objective situation, where they have experienced "discrimination" because it keeps them away from social contact [14]. Meanwhile, the psychological burden is a subjective experience that arises when they feel that what they have done and will do does not meet their expectations [22], and this is always connected to a poor mental condition [23]. Having feelings of "discrimination" in the context of social interaction means that they are very isolated and far from meaningful relationships, and this closes off opportunities for social contact with other people, so they are far from feeling happy [24], and in some sufferers with chronic illnesses, it is always related to low social skills [25], personality [26], unpleasant social interaction experiences [27], and low social support [28]. Thus, stigma and psychological burden are often related to each other [29].

In contrast, the results of qualitative studies have reported that some people living with tuberculosis feel protected from "discriminatory" behavior through a close relationship with their family, so that they can express their feelings and have the opportunity to discuss them with their family [30], but they feel helpless when facing stigma in society, as a result of the nature of tuberculosis which shows the characteristics of a disease that tends to be destructive and damaging [14, 31]. Meanwhile, stigma against people living with tuberculosis limits social interactions with the general public, and this can cause a situation similar to restrictions on movement and time, which in turn causes mental stress and influences efforts to seek health help [32]. This situation has been described as “social distancing” restrictions. However, to get closer to others, "physical distance" should still be encouraged because social interaction is still possible even though physically separated, and opportunities for direct interaction provide more opportunities to express feelings satisfactorily [33].

There is a dearth of qualitative research related to tuberculosis stigma. Several studies have highlighted how appropriate qualitative data can reveal aspects of the experience of tuberculosis stigma overlooked by clinical and epidemiological studies. It appears that the psychological burden may not increase during the initial phase of suffering from tuberculosis. However 45.5% of Ethiopian adults indicated that they frequently experienced symptoms of psychiatric morbidity in the 2020 measurement [34]. The relatively small number of qualitative studies related to tuberculosis stigma also shows that psychological burden is a very important concern in this research. Psychological burden is also the biggest challenge in mental health, this is because it is the gateway to emotional mental disorders, and if not addressed immediately, causes more severe mental problems, as is the result of a qualitative survey report on Russian teenagers suffering from tuberculosis [32]. In addition, in surveys and focus group studies of people living with chronic tuberculosis, 10.9% have reported experiencing prolonged mental stress [17].

Meanwhile, the course of mental stress is getting worse due to social stigma and self-stigmatization which describes increasingly severe physical and emotional mental conditions [9]. The risk of experiencing mental distress is frequently discussed in research on older adults and clinical populations. In studying the impact of tuberculosis stigma resulting from not having a life partner [35], stigma was a major theme and mental distress was identified in qualitative studies. In addition, for people who died in urban slum areas, the stigma of Tuberculosis as a cause of death became a central issue that developed in society [36]. It is increasingly clear that stigma and psychological burden are important concerns during tuberculosis treatment. Given that psychological burden is common throughout the life span of tuberculosis sufferers [37], a life course approach that examines the psychological burden in individuals with tuberculosis is appropriate. However, based on our observations, there has been no research that specifically explores the psychological burden in the context of tuberculosis stigma using qualitative methods, so this research aims to explore coping efforts when people living with tuberculosis feel the psychological burden due to stigma in society.

## Method

### Design

This research used a qualitative fenomenologies design, and face-to-face semi-structured interviews were conducted to explore the experiences of tuberculosis sufferers regarding obstacles, psychological burdens due to social stigma in society, and coping efforts made in society. This was done to gain valuable knowledge about their experiences while living with Tuberculosis [38], and researchers believe that the resulting data can offer complete and universal truth to explore their experiences while living with Tuberculosis [39]. Meanwhile, this research reporting guide uses the consolidated criteria for reporting qualitative research checklist (COREQ) [40].

### Methodological approach

The Reflexive thematic analysis recommended by Braun and Clark was to analyze the data and identify themes and patterns in the data to answer research questions about their experiences [41]. This is done with the hope that this method can answer the research objectives that have been determined previously. These themes were generated through reflexive and ongoing engagement with the data through the analysis process conducted by the researcher [42]. The resulting data was taken from the experiences of people living with tuberculosis which is in line with the critical realist framework of research, so this analysis is based on data and not theory as a whole [43].

### Participant

Participants were recruited using a purposive sampling technique of 25 participants. The characteristics of age, gender, age, level of education, work, monthly income, smoking, history of a family member with Tuberculosis, and number of family members in one household were considered in sampling. A diverse and representative sample that reflects the population of family members is drawn to obtain meaningful perceptions and experiences. The individual criteria in this study have been determined, namely (1) people living with tuberculosis who are still actively seeking treatment, (2) do not have severe comorbidities (heart failure, COPD), (3) live at home with their family, (4) willing to be a participant. Braun and Clarke’s assessment was used for the sampling strategy [44]. Determining the sample size used data saturation, and data saturation was achieved at the 23rd interview because no new information emerged [45]. Next, two more participants were interviewed to check new information. Data saturation and the final sample were then confirmed by the 25th participant. More details can be seen in Table 1.

**Table 1:** Characteristics of participants living with tuberculosis.

NO	Participant characteristics	n	Percentage
1	Gender		
	Male	16	64%
	Female	9	36%
	Age		
	20–29	1	4%
	30–39	7	28%
	40–49	4	16%
	50–59	8	32%
	˃ 60	5	20%
2	Level of education		
	Elementary school	10	40%
	JUNIOR HIGH SCHOOL	9	36%
	SENIOR HIGH SCHOOL	4	16%
	Diploma / Bachelor’s degree	2	8%
3	Work		
	Home Worker	14	56%
	Unemployment	3	12%
	Private sector employee	6	24%
	Civil Servants/TNI/Polri	2	8%
4	Monthly income		
	500.001–1.000.000	8	32%
	1.000.001–2.000.000	9	36%
	2.000.001–3.000.000	3	12%
	≥ 3.000.000	5	20%
5	Smokes		
	Yes	13	52%
	No	12	48%
6	History of a family member with Tuberculosis		
	There is	13	52%
	There isn’t any	12	48%
7	Number of family members in one house		
	2	5	20%
	3	7	28%
	4	10	40%
	≥ 5	3	12%

### Procedure

Researchers conducted in-depth face-to-face interviews with tuberculosis sufferers in a semi-structured manner while still paying attention to health protocols such as maintaining an ideal communication distance and using masks. The researcher conducted individual interviews to better understand her experiences, in line with what I researched [46]. Initiating interviews, informed consent, and socio-demographic data were collected. A thematic interview guide and researcher-developed field notes were used to record and identify verbal and non-verbal responses with open-ended questions developed by the researcher (Box 1), including the opening question “How are your relationships with the people around you”. Interviews were conducted at home in 2023 for 2 months, starting in August 2023 and ending in September 2023 (after obtaining ethical approval), conducted for a minimum of 25 minutes and a maximum of 40 minutes with an average of 35 minutes, and only the parties involved in interviews, namely researchers and participants only, using tools in the form of field notes and data recording devices. The interviewers for this research were 2 researchers, a senior lecturer with a mental nursing specialist orientation on psychosocial issues, and a PhD from the Vocational Faculty of Airlangga University. The other interviewer was a senior lecturer in Medical-Surgical Nursing with a Masters in Health. All participants were interviewed and observed in two rounds by the researcher directly, namely the first round to identify their experiences, and the second round to clarify topics that were still poorly understood to obtain valid data. Indonesian was used in this interview, and an interview guide was used to remind researchers of the topic of discussion and ensure that all main topics had been discussed, including a discussion of the psychological burden felt while living with tuberculosis and how coping efforts were made through interactions with the environment. The results of individual interviews were transcribed and confronted with nonverbal responses through field notes for data analysis and review to increase data accuracy [45]. The interview was stopped if no new data perspectives were found and it was deemed that the data search had reached data saturation.

**Box 1. Interview guide**

How is your relationship with those around you

How do you feel when interacting with the community (at home, socializing in the community, and seeking treatment at the clinic)

What is your attitude when interacting with society?

What are your hopes for all parties regarding the illness you are experiencing?

What were your obstacles during the treatment process?

How do you behave when your environment treats you differently than you expect?

What do you do to feel comfortable?

Is there anything else you wish to feel happy about while undergoing this treatment?

### Reflexivity

All interviews were transcribed verbatim, and labeled with participant codes. Interviewers simultaneously transcribed all data, allowing for consistent reflection and exploration of new ideas. The transcription was adjusted to Indonesian and matched with the audio recording to ensure accuracy. Data analysis used the Interpretative Phenomenology Analysis approach [47]. Following the data analysis guide from Braun and Clarke, 2019 [42], interview transcripts and field notes were read carefully to identify emerging themes, and then categorize important words that were related to each other. Researchers read the text as a whole and try to understand the overall meaning and develop keywords and concepts through dialogue with the text. Researchers also maintain openness through efforts to reflect on varying interpretations to monitor assumptions and biases through a triangulation process with participants [45]. Next, themes are interpreted from the components of the experience to the entire experience and back again. The researcher seeks to gain an understanding and engagement with texts related to the phenomenon under study. Finally, each sentence is analyzed and confronted with the data in the notes field, and through this process, important themes are discovered. These themes were then reconstructed into a description of the experiences experienced by people living with tuberculosis regarding the psychological burden due to social stigma and how they interact to achieve adaptive coping [48].

### Ethical considerations

Research procedures were carried out following the principles of the Declaration of Helsinki and were approved by the Research Ethics Committee of Muhammadiyah Lamongan University (number: 329 / EC /KEPK–S3 / 08 / 2023, dated August 1, 2023. All participants gave their consent and were informed that they could withdraw from the research any time. Informed consent was obtained from each research participant to publish their responses while maintaining anonymity, and the place and time of the interview were arranged to maintain privacy and confidentiality. Respondents’ identities were anonymized to maintain confidentiality.

## Results

Individuals from a variety of age groups and life situations were interviewed, so that the findings may reflect a variety of developmental contexts and environments in which powerlessness is felt. Therefore, this research is the first qualitative research that explicitly focuses on the psychological burden felt due to social stigma from society. Qualitative analysis of semi-structured interviews produced four themes relevant to the research questions, namely (1) Perception of stigma limiting space and time, (2) Opportunities for interpersonal interaction become narrow, (3) mental stress as a challenging emotion, (4) Expanding coping efforts.

### The perception of stigma limiting space and time

Feelings of depression when limiting physical distance and avoiding social contact with the community due to suffering from Tuberculosis are consequences that must be accepted from the loss of direct interaction. On one occasion they wanted to meet face to face to socialize with the community, but they lacked confidence in carrying themselves, they expressed their feelings:

I am like people in general, I want to be actively involved in society, but my disease (tuberculosis) makes me very inferior (male, 45 years old, P-04)

Another said

I had to hold back my cough when I was examined at the polyclinic, and I had to hold back my coughing response until I left the room, and that made me feel tired because of holding in my cough for too long (Male, 35 years old, P-18)

Meanwhile, their physical condition (people living with tuberculosis) does not allow them to be actively involved in carrying out daily activities.

It feels like if I work a little harder, my breathing feels like it’s just in my neck, this could be because I’ve been resting for too long or my illness causes me to get tired easily (male, 58 years old, P-21)

In the course of their lives, several participants also expressed their disappointment due to restrictions on physical distance in social interactions, because they were not given enough space and time to meet and meet face to face.

When I first suffered from this disease (tuberculosis), my emotions were very excessive, sometimes I cried alone or even my emotions exploded because on several occasions the people around me shortened the time they spent talking to me, and my illness (tuberculosis) was an obstacle. (male, 57 years old, P-16)

Likewise, other participants expressed that they felt the need to look people in the face when interacting with other people, so as not to be seen as haughty and arrogant, but some people they met always behaved as they had always done before, always avoiding eye-level contact, and this made them very disappointed, this is how their disappointed response looked like:

What I can’t accept is when people look away when talking to me, it feels like I don’t have enough respect… even doctors are like that sometimes, when they examine me, I’m told to look to the right or left, that’s what makes me sad. How contagious is my disease? (tuberculosis) (male, 51 years old, P-25)

The partition to prevent infection aggravated their mental pressure, like this, they expressed their feelings:

The obligation to wear a mask makes it clear to people’s opinion that I have been identified with this disease (tuberculosis), and this makes me embarrassed and insecure. (male, 36 years old, P-07)

### The opportunities for interpersonal interaction become narrow

On several occasions interaction with family is very necessary, at least to strengthen ties of brotherhood, but this does not apply to people living with tuberculosis, as the man’s parents complained.

I want to meet and have a casual chat with my child who hasn’t been home for a long time because of college, but my wife doesn’t allow me to be close to my child, because she’s afraid my child will be infected… I’m only allowed to contact her via cell phone…I lost direct contact with my child (female, 57 years old, P-06)

Meanwhile, social stigma cannot be removed from the minds of people around people living with tuberculosis, so they feel lonely.

My wife forbids me from serving shop customers, even though my wife is very busy, so my wife would rather the shop close than me help serve customers… my wife is very protective of my illness, which is good… so my only activity is watching TV. rather than loneliness (male, 39 years old, P-09)

And other similar things were also expressed when there was a Thanksgiving event at a neighbor’s house:

I wanted to attend the Thanksgiving invitation for the bride and groom of my brother’s son but was prevented by my wife. My wife was very worried that my disease (tuberculosis) would get worse because there were several guests present who might have the disease. The same disease as me…my wife is right, so I get well quickly, but it seems like I don’t get along with my sister (female, 32 years old, P-17)

Likewise children’s attitudes towards their parents who suffer from Tuberculosis. His protective attitude towards his parents made them lose close friends, this is the story.

Since my son took me to a neighboring village for treatment, I haven’t had any friends… it feels like I’m in prison. Maybe this is their way of being devoted to me as the mother who raised them (female, 60 years old, P-08)

And others

I was very frustrated, I felt confined and couldn’t leave the house… the treatment schedule was still 1 month away… it felt like that time was too long (male, 32 years old, P-14)

Until they feel pressured in their activities, they express their complaints like this:

This disease (tuberculosis), means that I am not very free to hang out with friends (male, 21 years old, P-02)

### The mental stress as a challenging emotion

In conditions that are constrained to avoid cross-infection, at the same time many emotions challenge us not to get involved in prolonged sadness. While a wife protects her phlegm to prevent infection, this is how a husband expresses his feelings:

At first, I was very angry when I coughed and my wife in a loud tone told me to throw the phlegm into the toilet and flush it many times… I just believe, my wife might not be wrong… maybe it’s better to avoid infecting other people (male, 53 years old, P-24)

Experiences of stigmatization and challenging emotions also emerged as part of how they increased hope and confidence in healing their illness. Thus physical distance is a barrier to being able to communicate with other people, providing fewer opportunities to meet, and making them more realistic in viewing the environment, thus strengthening the assumption that they understand other people’s attitudes, as they argue.

At first, I was very sad when people looked away when they met me in person… but I realized that they were afraid of getting infected… it was their part to protect themselves from getting infected, and not to insult me.…this is what made me obey all the messages from health officials so as not to make people afraid because of me (female, 49 years old, P-20)

And another said

I was advised by the doctor not to meet face to face when talking to other people, even though the person I was talking to was suffering from an illness like me.. that’s a health protocol… I have to obey and not feel disturbed (male, 37 years old, P-15)

Participants revealed several ways to divert attention due to the boredom they experienced when they were still prohibited from interacting with other people due to health protocols to prevent the transmission of droplet infections. Like this, they divert attention

Every morning I go to the 2nd floor of my house, and always do deep breathing exercises as taught by the health worker… this makes me feel less anxious (male, 32 years old, P-15).

Other participants also said

This guide on how to cough effectively has become my comfort, every time I have time to practice it. Apart from coughing, there is also a feeling of relief in my mind (male, 49 years old, P-23)

Meanwhile, with full humility, some people living with tuberculosis take a spiritual-religious approach to be calm and happy, and believe that everything is God’s will, as they surrender,

I am sure I will recover… I have fulfilled all the conditions recommended by health workers… God is most merciful and most merciful towards his servant (male, 58 years old, P-10)

Other participants also considered the disease to be a test from God and not a cause of death, as they believed

This disease is not a means to die… this disease is a trial from Allah… I am willing to go through it… Allah in giving trials will not exceed the ability of His servants… I hope this will pass soon, and I will recover completely (male, 57 years old, P-05)

### Expanding coping efforts

There is a strong desire to change the situation, and overcoming stressful conditions becomes a challenge to get out of misery, with several alternative problem solutions,

Honestly, I am a person who is very familiar with my neighbors… I like to chat and chat with my neighbors, but at this time there is no one to blame, why should I submit to my child not to meet other people first. It’s better not to communicate with other people, it’s not that I don’t want to interact with people around me, but preventing infection is what I prioritize…let me do it and hopefully not someone else…and I will recover quickly (female, 53 years old, P-24)

The same thing was also expressed by other participants

Other people don’t want to meet me face to face, but I think it’s normal.. indeed my disease is very contagious, and health workers say that tuberculosis is transmitted through breathing… I don’t feel tormented or offended (male, 57 years old, P-05)

Meanwhile, to be able to recover completely, obeying all the recommendations of health workers, especially routine treatment and wearing masks, is something they always do consistently, according to their comments.

The mask that I wear is my mainstay weapon to prevent contracting and not contracting my disease…this is what I often hear from health workers (Male, 38 years old, P-22)

And others

I understand how important the medicine I take is… I consider the need to take medicine regularly and seek regular treatment as conveyed by health workers as a challenge… It’s true, sometimes I feel nauseous after taking medicine… I need the medicine… the need for me to recover quickly (female, 49 years old, P-11)

## Discussion

The psychological burden felt by people living with tuberculosis is an important discussion in this research because the perceived stigma ultimately influences their flexibility in determining adaptive coping strategies [49]. It aims to understand how people living with tuberculosis endure the psychological burden when the perceived stigma outweighs the disease. This is an important context for studying psychological burdens because it has not occurred before in Indonesia. This study, unlike any previous study, has highlighted the inevitable impacts experienced by people living with tuberculosis. This emphasizes how perceived societal stigma can disrupt their emotions and social interaction patterns [50], thus preventing them from being actively involved in interpersonal relationships [51]. This situation requires them to choose and organize appropriate coping strategies [20].

Likewise, other research has indicated that tuberculosis is felt to be very discriminatory by society, especially the stigma as a very contagious disease [52], and for that reason, people distance themselves from socializing with people infected with Tuberculosis [53], and this can be a very painful experience for people living with tuberculosis [54]. In line with previous research reports [20], overall, this research also emphasizes the experiences felt by people living with tuberculosis, and how societal stigma creates a psychological burden, thereby preventing them from being actively involved in social interactions.

The discriminatory behavior felt by some people living with tuberculosis due to widespread societal stigma, becomes an additional characteristic and experience when carrying out social interactions in society [55], because they do not have the opportunity to communicate and interact with society, as reported in this study, and this was not the main target in several previous studies. They only focus on strict management of tuberculosis transmission to avoid cross-infection [56]. Several studies have also studied how to protect yourself, starting to use a mask every day [57–60], limiting yourself to social contact [60], special site management for sputum [61, 62], so they are preoccupied with clinical protocols.

Even though people living with tuberculosis are forced to carry out strict cross-infection prevention management amidst stressful situations due to perceived stigma, this also requires readjustments to create a comfortable, peaceful, and prosperous mood [60, 63, 64]. Prioritizing the psychological side effects of people living with tuberculosis, this research emphasizes the impact of stigma which affects their emotional mentality, interaction patterns that develop, and perceived obstacles, so that throughout their lives, they are required to make coping efforts through the use of coping flexibility to achieve adaptive coping.

Regarding the feelings experienced by people living with tuberculosis, this study reports the psychological burden felt due to societal stigma which continuously attacks their mental health, as was also reported in previous research [65]. Research results that report feelings of shame, depression, anxiety, and helplessness experienced by people living with tuberculosis [66, 67], because community stigma is felt to be very painful, strongly support these findings. For this reason, people living with tuberculosis should explore the coping resources that can be used, to make them feel strong, resourceful, and flexible and use the coping resources they have to resolve the mental stress they feel [15, 68–70]. Apart from that, other studies also report that discriminatory behavior due to public stigma aimed at TB sufferers is used as a trigger to increase self-esteem [71]. This makes them challenged to solve their problems, in the end, they have great hopes for achieving prosperity [72], and they must realize that the stigma against tuberculosis does exist, so it is important to expand the identification of the resources they have [73].

A finding that should receive serious attention, such as the current findings, is that the psychological burden described by participants captures the perceived inferiority of social interaction processes that rely on intrapersonal interactions compared to interpersonal ones. There is momentum behind using the terminology of physical distance because this approach is done to oneself in the interaction process [74], and increasing confidence through self-management is a communication exercise with oneself that must be carried out to get a therapeutic effect [75]. Bearing this in mind, it is very interesting that the participants in this study positioned interpersonal interactions as still inadequate. Even his personal experience, which is used as a measure to overcome the psychological burden he is experiencing, has not been able to overcome the psychological burden caused by suffering from tuberculosis. This is in line with research reports, that intrapersonal interaction does not improve their mood [76], compared with interpersonal communication [77], and through communication with other people, some of them can resolve the physical burden due to tuberculosis which can reduce their psychological stress [78–80], they even get the opportunity to express their problems more widely [81, 82].

The findings reported in the current study also illustrate that the loss of social interaction contributes to the psychological burden felt due to societal stigma haunting the minds of people living with tuberculosis. However, several studies have reported that some people living with tuberculosis still have the opportunity to interact with officers. when seeking treatment [83, 84] and with family at home [30], but cross-infection must be watched out for when interacting with them because tuberculosis is transmitted through droplet infection, where direct contact is very facilitative for the transmission of tuberculosis germs [85–87]. This interaction problem may have contributed to the psychological burden felt, because they had difficulty expressing their feelings due to the social distance that limited them, considering that several participants expressed the protective nature of previous experiences of perceived stigma. However, being used to being stigmatized by society makes physical distance less of a challenge [88, 89], and the perceived psychological burden may not generally increase during the treatment of societal stigma, but the loss of interaction with social media can have an impact on the psychological burden felt. According to the 2020 Global Tuberculosis Report, loss of social interaction can cause psychological and emotional harm to some people [90]. Thus, the psychological burden resulting from the loss of social contact is felt by individuals of various ages.

Although several research reports have identified the economic burden of people living with tuberculosis [91–93], this is not commensurate with the mental pressure they face [94, 95], as reported in this study. There is a strong desire among them to identify and use all the coping resources they have amidst the psychological burden they feel as a result of suffering from tuberculosis, and this is a very interesting finding to discuss. This finding is very relevant to their efforts to find a way out of their misery so that they can survive sporadic mental attacks [96, 97], and this is obtained when their psychological environment can explore personal resources and environmental resources to free themselves from feelings of depression [98–100], and a religious spiritual approach is the most ideal approach [101, 102] as also reported in this research. Therefore, they have a sense of self-confidence and physical strength that makes them feel protected, fulfilled, and satisfied [88, 103]. Thus, they feel that nothing cannot be resolved, because pressure turns into a challenge [104]. Apart from that, to support the expansion of identification of coping sources in overcoming mental stress to meet the psychological needs of complex problems that must be resolved, tuberculosis sufferers need to have a very high fighting spirit which is supported. with a strong desire to recover [105, 106], to be free from feelings of sadness and misery [107]. Don’t forget, that they also always pray to God Almighty asking for His mercy to cure the illnesses they suffer from, and this is also often done by people who suffer from other chronic illnesses [108].

In contrast, there are not many other research reports that reveal that people with tuberculosis, especially in very severe conditions, can identify and expand their coping resources, and hopelessness is a feeling they often express [17]. However, several studies on this response have discussed the benefits obtained from using some of the coping resources they have to make them feel protected from mental stress, and these are used as a buffer from the psychological burden they feel [109]. Several research findings have also agreed that physical and psychological pressure on people living with tuberculosis can undermine their hopes [54], and this requires effective coping strategies to ensure their well-being [110].

Research reports have shown that some people living with tuberculosis have a strong hope of avoiding the mental stress caused by the stigma of tuberculosis, and the avoidance pattern is the one they most often choose, while still adhering to the treatment regimen. In this case, the use of avoidance patterns which are coping resource attributes of the emotional focus of coping is used to ensure their well-being [80], as is also used in people living with HIV-AIDS [111, 112] and people living with leprosy [113, 114], and for sufferers of other chronic diseases, based on the results of meta-analysis studies, “avoid” is used for someone who experiencing physical and emotional exhaustion [115]. However, in this study, avoidance has a positive meaning in preventing transmission. Therefore, coping that focuses on problems and focuses on emotions is an attribute for solving problems, and a coping approach that focuses on emotions should be used as a temporary approach to be free from pressure [116]. Then problem-focused coping is used to solve problems so that TB sufferers are free from physical problems which are a source of pressing stressors [117].

As in this study, living with tuberculosis is felt very differently, because it is considered a disgusting disease [68], and this feeling can reduce motivation to seek treatment, thereby stimulating efforts to use maladaptive coping alternatives [21]. Thus, people living with tuberculosis cannot avoid stigmatizing and discriminatory behavior by society, which can disrupt their self-concept [20], and this requires several coping alternatives to respond to the various stresses they experience [118, 119].

People living with tuberculosis need to develop adaptive coping efforts to alleviate their suffering and meet the ultimate goal of the treatment they are undergoing, namely achieving optimal recovery without enduring heavy stress that can burden them mentally [96].

Meanwhile, believing that tuberculosis can be cured is their effort to motivate themselves to undergo a treatment program, as also reported in other research [120]. Therefore, as in the findings of this study, some people living with tuberculosis want a positive solution to be free from misery, and regular treatment until they have a complete recovery status is the main goal they want to eliminate the burden of suffering [121].

By expanding their identification of various sources of coping, they have many choices and use them. In this case, people living with tuberculosis need to learn, practice, and develop themselves which can be integrated as a coping effort in dealing with their problems [121]. Through the skill of selecting adaptive coping sources, people living with tuberculosis must become individuals who are flexible, not rigid, open, and have a broader vision and mission, so that they have high flexibility to use alternative coping methods that suit the situation and conditions [118], both now and in the future. This is also what people living with tuberculosis must adapt to the environment in which they live to achieve optimal recovery [21].

## Limitations

The characteristics of the participants (tuberculosis sufferers) are different, of course, their perceptions and ways of expressing their experiences will also be different so they have great difficulty in expressing their feelings. They also have difficulty expressing how to solve problems in a meaningful way. Even though there are limitations in obtaining accurate data, thanks to the researcher’s therapeutic communication skills, the participants felt comfortable during discussions with the researcher, including feeling comfortable in expressing feelings, obstacles in interacting in society, and efforts made to solve problems. Even though the disease they experienced was highly stigmatized in society, they revealed in detail the problems they faced and how they reached solutions to achieve adaptive coping. Therefore, for qualitative researchers, giving participants space and time to express their feelings without judgment is important so that they can freely express their feelings without hiding them, so that natural data is obtained, appropriate to the context being studied [122].

Data analysis was carried out based on the data found, this sometimes found bias between themes and supporting data, but thanks to the triangulation process with qualitative research experts, tuberculosis eradication program holders, and confrontation of unclear data with participants, it was able to produce a better understanding. contribute experiences and perceptions through a professional approach.

## Conclusion

This research highlights the psychological burden felt when people living with Tuberculosis experience highly discriminatory treatment due to social stigma in society. These findings show that the societal stigma they receive is very disturbing mentally and emotionally which can cause tension along with the psychological burden they feel, so they are motivated to expand their coping efforts. Given the possibility that stigma and discrimination persist during tuberculosis treatment programs, it is important to consider the psychological burden in this context, both on the general population and on groups affected by stigma.

## Supporting information

S1 File The supporting information file. (DOCX)
