# Supplementary material for: Coping efforts made: Psychological burden of people living with tuberculosis due to social stigma in society. A qualitative phenomenology study
Source: PLoS One. 2024 Jul 30;19(7):e0303331. doi: 10.1371/journal.pone.0303331 (PMC11288456; doi:10.1371/journal.pone.0303331)
Supplement: S1 File — (DOCX) [file pone.0303331.s001.docx]

**Research result**

***The Perception of stigma limiting space and time***

Feelings of depression when limiting physical distance and avoiding social contact with the community due to suffering from Tuberculosis are consequences that must be accepted from the loss of direct interaction. On one occasion they wanted to meet face to face to socialize with the community, but they lacked confidence in carrying themselves, they expressed their feelings:

I am like people in general, I want to be actively involved in society, but my disease (tuberculosis) makes me very inferior (male, 45 years old, P-04)

Another said

I had to hold back my cough when I was examined at the polyclinic, and I had to hold back my coughing response until I left the room, and that made me feel tired because of holding in my cough for too long (Male, 35 years old, P-18)

Meanwhile, their physical condition (tuberculosis sufferers) does not allow them to be actively involved in carrying out daily activities.

It feels like if I work a little harder, my breathing feels like it's just in my neck, this could be because I've been resting for too long or my illness causes me to get tired easily (male, 58 years old, P-21)

In the course of their lives, several participants also expressed their disappointment due to restrictions on physical distance in social interactions, because they were not given enough space and time to meet and meet face to face.

When I first suffered from this disease (tuberculosis), my emotions were very excessive, sometimes I cried alone or even my emotions exploded because on several occasions the people around me shortened the time they spent talking to me, and my illness (tuberculosis) was an obstacle. (male, 57 years old, P-16)

Likewise, other participants expressed that they felt the need to look people in the face when interacting with other people, so as not to be seen as haughty and arrogant, but some people they met always behaved as they had always done before, always avoiding eye-level contact, and this made them very disappointed, this is how their disappointed response looked like:

What I can't accept is when people look away when talking to me, it feels like I don't have enough respect... even doctors are like that sometimes, when they examine me, I'm told to look to the right or left, that's what makes me sad. How contagious is my disease? (tuberculosis) (male, 51 years old, P-25)

The partition to prevent infection aggravated their mental pressure, like this, they expressed their feelings:

The obligation to wear a mask makes it clear to people's opinion that I have been identified with this disease (tuberculosis), and this makes me embarrassed and insecure. (male, 36 years old, P-07)

***The Opportunities for interpersonal interaction become narrow***

On several occasions interaction with family is very necessary, at least to strengthen ties of brotherhood, but this does not apply to Tuberculosis sufferers, as the man's parents complained.

I want to meet and have a casual chat with my child who hasn't been home for a long time because of college, but my wife doesn't allow me to be close to my child, because she's afraid my child will be infected... I'm only allowed to contact her via cell phone. ..I lost direct contact with my child (female, 57 years old, P-06)

Meanwhile, social stigma cannot be removed from the minds of people around Tuberculosis sufferers, so they feel lonely.

My wife forbids me from serving shop customers, even though my wife is very busy, so my wife would rather the shop close than me help serve customers... my wife is very protective of my illness, which is good... so my only activity is watching TV. rather than loneliness (male, 39 years old, P-09)

And other similar things were also expressed when there was a Thanksgiving event at a neighbor's house:

I wanted to attend the Thanksgiving invitation for the bride and groom of my brother's son but was prevented by my wife. My wife was very worried that my disease (tuberculosis) would get worse because there were several guests present who might have the disease. The same disease as me...my wife is right, so I get well quickly, but it seems like I don't get along with my sister (female, 32 years old, P-17)

Likewise children's attitudes towards their parents who suffer from Tuberculosis. His protective attitude towards his parents made them lose close friends, this is the story.

Since my son took me to a neighboring village for treatment, I haven't had any friends... it feels like I'm in prison. Maybe this is their way of being devoted to me as the mother who raised them (female, 60 years old, P-08)

And others

I was very frustrated, I felt confined and couldn't leave the house... the treatment schedule was still 1 month away... it felt like that time was too long (male, 32 years old, P-14)

Until they feel pressured in their activities, they express their complaints like this:

This disease (tuberculosis), means that I am not very free to hang out with friends (male, 21 years old, P-02)

***The mental stress as a challenging emotion.***

In conditions that are constrained to avoid cross-infection, at the same time many emotions challenge us not to get involved in prolonged sadness. While a wife protects her phlegm to prevent infection, this is how a husband expresses his feelings:

At first, I was very angry when I coughed and my wife in a loud tone told me to throw the phlegm into the toilet and flush it many times... I just believe, my wife might not be wrong... maybe it's better to avoid infecting other people (female, 53 years old, P-24)

Experiences of stigmatization and challenging emotions also emerged as part of how they increased hope and confidence in healing their illness. Thus physical distance is a barrier to being able to communicate with other people, providing fewer opportunities to meet, and making them more realistic in viewing the environment, thus strengthening the assumption that they understand other people's attitudes, as they argue.

At first, I was very sad when people looked away when they met me in person... but I realized that they were afraid of getting infected... it was their part to protect themselves from getting infected, and not to insult me. ...this is what made me obey all the messages from health officials so as not to make people afraid because of me (female, 49 years old, P-20)

And another said

I was advised by the doctor not to meet face to face when talking to other people, even though the person I was talking to was suffering from an illness like me.. that's a health protocol ... I have to obey and not feel disturbed (male, 37 years old, P-15)

Participants revealed several ways to divert attention due to the boredom they experienced when they were still prohibited from interacting with other people due to health protocols to prevent the transmission of droplet infections. Like this, they divert attention

Every morning I go to the 2nd floor of my house, and always do deep breathing exercises as taught by the health worker ... this makes me feel less anxious (male, 32 years old, P-15).

Other participants also said

This guide on how to cough effectively has become my comfort, every time I have time to practice it. Apart from coughing, there is also a feeling of relief in my mind (male, 49 years old, P-23)

Meanwhile, with full humility, some Tuberculosis sufferers take a spiritual-religious approach to be calm and happy, and believe that everything is God's will, as they surrender,

I am sure I will recover ... I have fulfilled all the conditions recommended by health workers ... God is most merciful and most merciful towards his servant (male, 58 years old, P-10)

Other participants also considered the disease to be a test from God and not a cause of death, as they believed

This disease is not a means to die ... this disease is a trial from Allah... I am willing to go through it ... Allah in giving trials will not exceed the ability of His servants ... I hope this will pass soon, and I will recover completely (male, 57 years old, P-05)

***Expanding coping efforts.***

There is a strong desire to change the situation, and overcoming stressful conditions becomes a challenge to get out of misery, with several alternative problem solutions,

Honestly, I am a person who is very familiar with my neighbors... I like to chat and chat with my neighbors, but at this time there is no one to blame, why should I submit to my child not to meet other people first. It's better not to communicate with other people, it's not that I don't want to interact with people around me, but preventing infection is what I prioritize...let me do it and hopefully not someone else...and I will recover quickly (female, 53 years old, P-24)

The same thing was also expressed by other participants

Other people don't want to meet me face to face, but I think it's normal.. indeed my disease is very contagious, and health workers say that tuberculosis is transmitted through breathing ... I don't feel tormented or offended (male, 57 years old, P-05)

Meanwhile, to be able to recover completely, obeying all the recommendations of health workers, especially routine treatment and wearing masks, is something they always do consistently, according to their comments.

The mask that I wear is my mainstay weapon to prevent contracting and not contracting my disease...this is what I often hear from health workers (Male, 38 years old, P-22)

And others

I understand how important the medicine I take is ... I consider the need to take medicine regularly and seek regular treatment as conveyed by health workers as a challenge ... It's true, sometimes I feel nauseous after taking medicine ... I need the medicine ... the need for me to recover quickly (female, 49 years old, P-11)
